# Boxcar Averaging Scanning Nonlinear Dielectric Microscopy

**DOI:** 10.3390/nano12050794

**Published:** 2022-02-26

**Authors:** Kohei Yamasue, Yasuo Cho

**Affiliations:** Research Institute of Electrical Communication, Tohoku University, 2-1-1 Katahira, Aoba, Sendai 980-8577, Japan; yasuocho@riec.tohoku.ac.jp

**Keywords:** scanning nonlinear dielectric microscopy, boxcar averaging, scanning probe microscopy, scanning near-field microwave microscopy, scanning microwave impedance microscopy

## Abstract

Scanning nonlinear dielectric microscopy (SNDM) is a near-field microwave-based scanning probe microscopy method with a wide variety of applications, especially in the fields of dielectrics and semiconductors. This microscopy method has often been combined with contact-mode atomic force microscopy (AFM) for simultaneous topography imaging and contact force regulation. The combination SNDM with intermittent contact AFM is also beneficial for imaging a sample prone to damage and using a sharp microscopy tip for improving spatial resolution. However, SNDM with intermittent contact AFM can suffer from a lower signal-to-noise (S/N) ratio than that with contact-mode AFM because of the shorter contact time for a given measurement time. In order to improve the S/N ratio, we apply boxcar averaging based signal acquisition suitable for SNDM with intermittent contact AFM. We develop a theory for the S/N ratio of SNDM and experimentally demonstrate the enhancement of the S/N ratio in SNDM combined with peak-force tapping (a trademark of Bruker) AFM. In addition, we apply the proposed method to the carrier concentration distribution imaging of atomically thin van der Waals semiconductors. The proposed method clearly visualizes an anomalous electron doping effect on few-layer Nb-doped MoS_2_. The proposed method is also applicable to other scanning near-field microwave microscopes combined with peak-force tapping AFM such as scanning microwave impedance microscopy. Our results indicate the possibility of simultaneous nanoscale topographic, electrical, and mechanical imaging even on delicate samples.

## 1. Introduction

Scanning nonlinear dielectric microscopy (SNDM) is a scanning probe microscopy (SPM) method using near-field microwaves and frequency modulation (FM) [1]. Owing to an exceptionally high sensitivity to local capacitance variation below the tip, this microscopy has versatile applications in science and engineering of dielectrics and semiconductors [2,3]. SNDM was originally devised for imaging electric anisotropy of dielectrics such as ferroelectric domains [4] and has the potential to become a key technology for ferroelectric probe data storage enabling Tbit/inch^2^ recording density [5,6]. The scope of applications has also extended to the nanoscale evaluation of semiconductor materials and devices, including dopant profiling in miniaturized transistors [7,8], imaging the stored charges in flash memories [9], carrier distribution imaging on SiC power transistors [10], amorphous and monocrystalline Si solar cells [11,12], and atomically-thin layered semiconductors [13,14]. SNDM and its potentiometric extension can show true atomic resolution in surface dipole imaging on a Si (111)-(7 × 7) surface [15,16] and single-layer graphene on SiC [17]. Furthermore, new classes of SNDM called super-higher-order SNDM [10] and time-resolved SNDM [18] have recently emerged, enabling local deep level transient spectroscopy [18,19] and local capacitance-voltage profiling for semiconductors [20,21].

Among the members of the SNDM family, SNDM combined with atomic force microscopy (AFM) has been of importance for various applications. So far, SNDM has been mainly combined with contact-mode AFM (C-AFM), which permits topographic imaging by maintaining the contact force between a microscopy tip and the sample surface during the lateral scan of the surface [22]. However, C-AFM has a well-known drawback of higher probability for damaging the tip and the sample because of strong lateral forces [23]. For instance, atomically-thin van der Waals materials such as graphene and few-layer MoS_2_ can be peeled off during imaging, in our experience [13]. The tip is likely to deform, especially when using an expensive ultra-sharp tip for attempting the improvement of spatial resolution. In such a case, we need to make significant effort to carefully optimize measurement conditions to suppress the probability of the tip deformation as much as possible.

One possible way to overcome these problems is to combine SNDM with intermittent-contact AFM (IC-AFM), following the development history of AFM [24]. There were several IC-AFM methods such as so-called tapping mode AFM [23], peak-force tapping (a trademark of Bruker) AFM [25], and force volume (a trademark of Bruker) imaging [26] differing by typical contact frequency. However, intermittent contact operation can cause a significant reduction of the signal-to-noise (S/N) ratio or an increase of measurement time in SNDM imaging [13,27]. This is because the signal from the SNDM channel is normally generated only when the tip is in contact with or in very close proximity to the sample surface [28]. Signal intensity is highest on the surface but rapidly decreases below the noise level as the tip moves slightly away from the surface. This indicates that the achievable S/N ratio of SNDM is basically limited by the total contact time for a given measurement time and, therefore, SNDM combined with IC-AFM (IC-SNDM) essentially has a lower achievable S/N ratio than that combined with C-AFM because of the shorter total contact time. In addition, as previously pointed out, an actual S/N ratio can be further reduced from the achievable level, unless the detection bandwidth of the SNDM signal is appropriately chosen for the given contact time [27]. In particular, SNDM combined with peak-force tapping AFM (PFT-SNDM) is beneficial but suffers from a much lower signal intensity than that combined with C-AFM (C-SNDM), because the bandwidth of the signal acquisition is hardly optimized for intermittent contact operations [13].

In order to improve the S/N ratio of PFT-SNDM, here, we apply the idea of boxcar averaging to PFT-SNDM. By significantly extending the consideration and results given in the previous brief report [29], we develop a more sophisticated theory of PFT-SNDM to quantitatively explain the S/N ratio of SNDM including C-SNDM and PFT-SNDM. We show that, by utilizing gated signal acquisition followed by averaging, the optimal S/N ratio can be achieved even for PFT-SNDM. The measurement parameters required to maximize the S/N ratio can be quantitatively determined by the developed theory. In addition, along with the practical aspects on the implementation of the proposed method, we experimentally demonstrate and discuss actual improvement in the S/N ratio by measuring a test semiconductor sample. Furthermore, we address a recent application of the proposed method to the imaging of the dominant carrier concentration distribution in atomically thin van der Waals semiconductors. As reported in greater detail in our recent paper [14], we were able to observe an anomalous doping effect on few-layer MoS_2_ by utilizing the proposed method. Because of the similar imaging mechanism, the idea presented here can also be applied to the optimization of S/N ratios in other scanning near-field microwave microscopy such as scanning microwave impedance microscopy (SMIM) [30] combined with peak-force tapping AFM [25].

## 2. Principle of SNDM and Combination with IC-AFM

**Figure 1:**
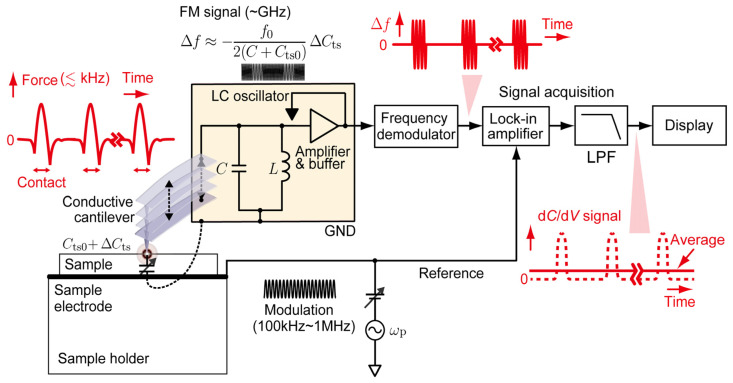
Schematic diagram of PFT-SNDM. The periodic contact of the tip with the sample surface generates a pulse train of frequency shift at the output of a frequency demodulator. The period and duty cycle of the pulse train are the same as those of the periodic contact. Because of the averaging effect on the pulse train in the signal acquisition, the signal intensity can significantly decrease. C-SNDM can be regarded as PFT-SNDM with a 100% duty cycle in terms of the S/N ratio.

SNDM is an FM based SPM method using an electric self-oscillator for sensing the variations in the capacitance between the conductive sharp tip and the sample [1]. A fingertip-sized LC oscillator oscillating in a gigahertz range is often employed as a self-oscillating capacitance sensor called a SNDM probe. Figure 1 shows a schematic diagram of SNDM [13]. Here, the diagram illustrates PFT-SNDM with vertical periodic cantilever motion for the later description but the explanation below also applies to C-SNDM except that the tip keeps in contact with the sample surface in C-SNDM. If the duty ratio in PFT-SNDM is defined as the rate of the contact time to the repeating period, C-SNDM can be regarded as PFT-SNDM with a 100% duty ratio in terms of S/N ratio. In SNDM combined with AFM, a conductive cantilever with a sharp tip is attached to the LC oscillator, which makes the tip-sample capacitance $C_{\mathrm{ts}}$ electrically connected in parallel with a built-in LC tank circuit. Because the variations in the tip-sample capacitance $\Delta C_{\mathrm{ts}}$ change the resonance frequency of the circuit, we can detect $\Delta C_{\mathrm{ts}}$ from the shift of oscillation frequency Δf from the center frequency $f_{0}$. In a typical condition, Δf is approximately proportional to $\Delta C_{\mathrm{ts}}$ with the proportionality constant of $-f_{0}/\left\{2\left(C+C_{\mathrm{ts}0}\right)\right\}$, as $\Delta C_{\mathrm{ts}}$ is several orders of magnitude smaller than the built-in capacitance C. $C_{\mathrm{ts}0}$ denotes the static component of $C_{\mathrm{ts}}.$ As a result of the relationship between Δf and $\Delta C_{\mathrm{ts}}$, the FM signal can be obtained by applying a sinusoidal modulation voltage across the tip and the sample. The modulation frequency is typically 10 kHz for C-SNDM and 100 kHz~1 MHz for PFT-SNDM. FM in SNDM can be normally regarded as narrow band FM because of the very low modulation index. We use a frequency demodulator in a microwave range for the demodulation of $\Delta C_{\mathrm{ts}}$ and a lock-in amplifier to obtain the first order capacitance variations, or a voltage derivative of capacitance, here called a d*C*/d*V* signal. The d*C*/d*V* signal is also called a $\varepsilon_{333}$ signal in the measurement of dielectrics, because the d*C*/d*V* signal is proportional to a nonlinear third-order dielectric constant described by a third-rank tensor [1]. The minimum detectable $\Delta C_{\mathrm{ts}}$ is typically as low as $2\times 10^{-22}\;F$ for a unity measurement bandwidth [16].

d*C*/d*V* signals arise from different mechanisms on different materials. One of the main mechanisms is an electric anisotropic property of a material such as ferroelectric polarization below the tip [1]. The polarity of a d*C*/d*V*, or $\varepsilon_{333}$, signal is inverted depending on the polarity of a ferroelectric domain. The second is the change in the depletion layer capacitance in a semiconductor material. As is often done using scanning capacitance microscopy [31], we can determine the polarity of dominant carriers, p- or n-type, on a local area of a semiconductor from the polarity of the d*C*/d*V* signal, and the signal intensity gives local information of carrier concentration with superior sensitivity. If reference samples for calibration can be prepared, the d*C*/d*V* imaging can be used for the nanoscale quantitative measurement of non-linear permittivity on dielectrics [32] and dopant profiling on semiconductors [8,12].

SNDM is combined with AFM for controlling the force between the tip and the sample and obtaining a simultaneous topographic image. Odagawa and Cho first demonstrated SNDM combined with C-AFM [22]. Recently, Yamasue and Cho integrated SNDM with PFT-AFM in a commercial SPM system (Bruker Dimension Icon, Billerica, MA, USA) to image atomically thin van der Waals semiconductors [13]. PFT-SNDM was able to visualize the distributions of dominant carrier concentration on atomically thin MoS_2_ including single-layer structures. In addition to the capability of avoiding damaging the tip and the sample, the imaging stability of the SNDM channel is improved by suppressing the probability of charge injection from the tip to the sample, which can accidentally cause abrupt changes to the signal intensity [14]. Another benefit is that PFT-AFM had much better reproducibility in the measurement of topographic height differences in different stacking layers, which helps the identification of the layer number. PFT-AFM also allows us to investigate the mechanical properties of sample [25], even on fragile biological samples [33,34]. Since SNDM is useful for imaging the local electric properties, its combination with PFT-AFM will permit the nanoscale investigation on the correlation between local electric and mechanical properties.

The problem is that PFT-SNDM has one order of magnitude higher contact frequency (typically at 2 kHz) than the typical frequency bandwidth of signal acquisition (~100 Hz). This results in much shorter contact time (~50 μs) than the typical time constants of signal acquisition (~1 ms). As shown in Figure 1, because the signal in the conventional PFT-SNDM is averaged through the signal acquisition regardless of whether the tip is in contact with the surface or not, that is, whether the signal is present or absent, an averaging effect drastically reduces signal intensity. The signal intensity decreased by a factor of the duty cycle (~0.1). The averaging effect can be avoided by gated signal acquisition and averaging with a tuned bandwidth, which enables a much higher S/N ratio for a given contact time and measurement time, as shown in the next section.

It is noted that, historically, the first IC-SNDM was developed by Hiranaga and Cho [24]. They proposed a measurement method that repeats a cycle of approaching, maintaining the contact, acquiring the d*C*/d*V* signal, and withdrawing for every measurement point. They reported that, compared to C-SNDM, their lab-made IC-SNDM achieved higher stability and better reproducibility in SNDM imaging on a ferroelectric material. The difference from PFT-SNDM treated in this paper is that the contact frequency and contact time in their method were about 25 Hz and 10 ms, respectively, which are two orders of magnitude lower and longer than those in PFT-SNDM. The contact time is taken long enough to wait for the signal to reach an intensity as high as that in C-SNDM, which results in a longer measurement time. In this case, no averaging effect occurred in the signal acquisition. Detailed discussion on S/N ratio has not been given in the literature. A similar IC-SNDM method has recently been implemented by Yamasue and Cho to a commercial SPM system utilizing force volume imaging rather than PFT imaging [27]. In their paper, they discussed the S/N ratio of IC-SNDM, which gave us a clue to the improvement of the S/N ratio by the boxcar averaging based scheme presented here. They pointed out that the balance between the bandwidth, or time constant of the signal acquisition, and the contact time of IC-SNDM needs to be optimized to obtain the highest S/N ratio in a given measurement time.

## 3. S/N Ratio of SNDM

**Figure 2:**
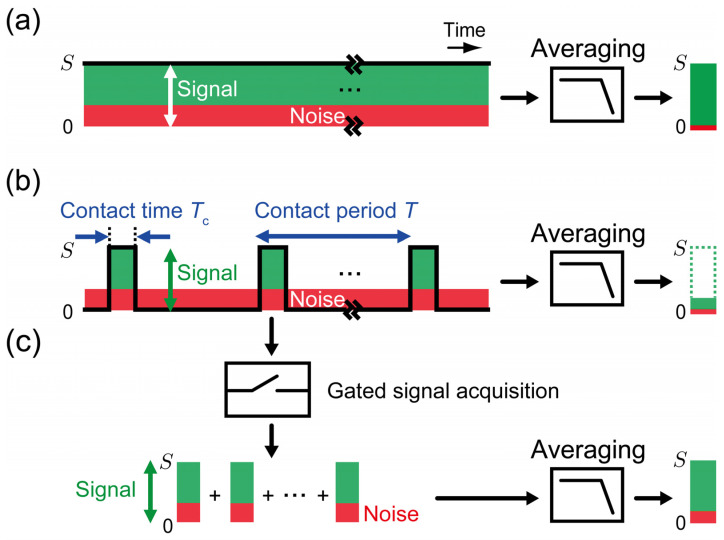
Schematic illustration of signal acquisition and the achievable S/N ratio in (**a**) C-SNDM, (**b**) PFT-SNDM, and (**c**) BA-PFT-SNDM. Green parts indicate the intensity of d*C*/d*V* signals, or the amplitude of frequency shift, in the capacitance sensor. Red parts depict noise levels.

In IC-SNDM, the intensity of a d*C*/d*V* signal typically becomes highest only when the tip is in contact with the sample but rapidly decreases below the noise level as the tip-sample separation increases. Therefore, the d*C*/d*V* signal is regarded as a pulse train with a duty cycle $D=T_{c}/T$, where $T_{c}$ and T denote the contact time and the contact period, respectively. A typical duty cycle in PFT-SNDM is as low as D=0.1, which has a significant impact on the conventional signal acquisition. Figure 2a,b compare the difference between a 100% duty ratio equivalent to C-SNDM and a low duty ratio corresponding to PFT-SNDM. For a 100% duty ratio, the signal continuously takes the highest level with no averaging effect, while the noise level decreases by a factor depending the detection bandwidth of the signal acquisition. On the other hand, in the case of the low duty ratio, the signal intensity decreases by a factor of D, as illustrated in Figure 2b. This is because the signal with a pulse train is averaged though continuous signal acquisition with a much narrower bandwidth than contact frequency. The noise level is the same as that in C-SNDM as long as the noise densities are the same regardless of whether the tip is in contact with the surface or not. Thus, PFT-SNDM has a significantly lower S/N ratio than C-SNDM.

The idea is to eliminate the periods that contain no signal but only the noise from the signal averaging stage. As shown in Figure 2c, we can utilize gated signal acquisition to extract the signal only when the tip is in contact with the surface. The extracted signal can be then regarded as a continuous signal like that in C-SNDM. The signal becomes suitable for averaging because the intensity remains highest. The idea here has been called boxcar averaging [35,36] and the proposed method here is called boxcar averaging PFT-SNDM (BA-PFT-SNDM). It is noted that the noise level becomes higher than that in C-SNDM because of the shorter total contact time for a given measurement time. In fact, we need to increase the bandwidth of a lock-in amplifier to acquire the signal at the highest intensity. If the bandwidth is tuned, the signal is expected to rise from the noise level to the highest level during contact time without causing the averaging affect. The increase of the bandwidth results in the increase of noise included in the output of the lock-in amplifier, while the noise level is further reduced through the subsequent averaging stage without reducing the signal intensity.

Here, we develop a theory to predict the optimal detection bandwidth and S/N ratio in BA-PFT-SNDM by extending the discussion given in the previous brief report [29]. To increase the level of the d*C*/d*V* signal acquired during contact, the output of a low-pass filter (LPF) in the lock-in amplifier needs to follow a pulse train of Δf signal fast enough and reach the vicinity of the highest value S, before the tip is away from the surface. Settling time and its methodology treated in a classical control theory help to describe this situation. Settling time $T_{S}$ is the time required for the output of a system to reach and stay within a given range in the vicinity of the final value after a step input. To maximize the signal level, $T_{S}$ needs to be smaller than $T_{c}$. In addition, $T_{S}$ should be smaller than $T_{p}$, which denotes the average time elapsed for constructing one pixel of a d*C*/d*V* image. If $T_{S}$ is determined for a given $T_{c}$ (or D) and $T_{p}$, we can calculate the minimum bandwidth of the signal acquisition required to obtain the highest signal intensity, which also minimizes the noise level. It is noted that the time constant of the LPF is related to $T_{S}$ but deviates from it in a high-order system like the LPF stage of a lock-in amplifier.

**Figure 3:**
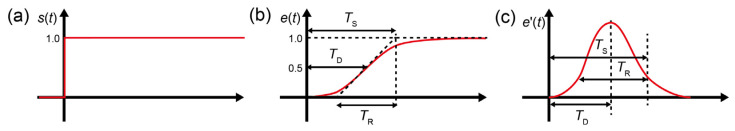
Step response of a system and the definition of settling time ($T_{S}$) based on the delay time ($T_{D}$ ) and the rise time ($T_{R}$ ). (**a**) step input (**b**) step response (**c**) impulse response. The step response [(**b**)] is given by integrating the impulse response [(**c**)] approximated by a Gaussian function.

We discuss the S/N ratio in IC-SNDM based on the settling time. However, to exactly obtain the settling time for a given specific system, it is necessary to derive the step response of the system, or the transient output response to a step input. Thus, we employ the traditional simple methodology proposed by Elmore to treat the transient output response of a linear system [37]. In this methodology, the impulse response of a given system, or the time-derivative of the step response, is approximated by the impulse response of a Gaussian transfer function system that can be mathematically more tractable. The approximation is justified because the impulse response of a linear time-invariant system becomes closer to that of a Gaussian transfer function system, as the order of the system increases, under reasonable assumptions. The impulse response of the Gaussian transfer function system becomes a Gaussian function of time again. This implies that we can approximate the step response of interest by the time-integration of the impulse response from the Gaussian transfer function. As shown in Figure 3, the step input to a signal acquisition system (Figure 3a) causes the step response with a settling time depending on the bandwidth (Figure 3b). The step response is given by the integration of impulse response approximated by the cumulative distribution function of the Gaussian function (Figure 3c). Thus, we can define the rise time $T_{R}$ by the cumulative frequency of the Gaussian function. Note that Gaussian transfer function systems are not causal. Unlike the original system of interest, the output of the corresponding Gaussian system arises before the input is applied. The impulse response already reaches the maximum at t=0. To adjust the time of the maximum output from the Gaussian system to that from the original system, a delay time $T_{D}$ is further introduced in this methodology. This methodology works well for the filters without significant overshoot in the step response, as realized in a typical LPF stage of a lock-in amplifier.

According to the work by Elmore, $T_{D}$ and $T_{R}$ are approximated using the impulse response of interest $e^{\prime }\left(t\right)$, respectively.

(1) $$T_{D}=\int_{0}^{\infty }te^{\prime }\left(t\right)dt,$$

(2) $$T_{R}=\sqrt{2\pi }\sqrt{\left(\int_{0}^{\infty }t^{2}e^{\prime }\left(t\right)dt\;\right)-T_{D}^{2}}$$

Equations (1) and (2) give the first order and second order moments of $e^{\prime }\left(t\right)$ when $e^{\prime }\left(t\right)$ is approximated by a Gaussian function in the time domain. The first and second order moments correspond to the average and variation of the Gaussian function, respectively. Then, we define the settling time as follows:

(3) $$T_{S}=T_{D}+\alpha \left(\gamma \right)\frac{T_{R}}{2},$$

where α denotes a constant depending on the parameter γ, which indicates how close to the maximum intensity the signal level is required to be at the settling time. γ is typically chosen to be γ=90% or 95% relative to the maximum intensity, or the final value of the output at the steady state. If the signal acquisition system is decomposed into *m* lower order cascaded systems connected in the series, it can be shown that $T_{D}$ and $T_{R}$ are given by the sum of the delay time and the root of the square sum of the rise time in each system.

(4) $$T_{D}=\overset{m}{\underset{i=1}{\sum }}T_{D_{i}},$$

(5) $$T_{R}=\sqrt{\overset{m}{\underset{i=1}{\sum }}T_{R_{i}}^{2}}.$$

To obtain the signal with the γ% level to the maximum intensity at the settling time, the following condition should be satisfied.

(6) $$T_{S}\leq T_{c}=DT.$$

In addition, we require $T_{S}$ to be smaller than $T_{p}$, denoting the time allowed for the tip to stay for one pixel of a d*C*/d*V* image.

(7) $$T_{S}\leq T_{p}.$$

In PFT-SNDM, we acquire a d*C*/d*V* signal from Δf by using a lock-in amplifier with a high order LPF. Let us assume that the filter consists of *m*-cascaded first-order filters with a time constant of $\tau_{1}$ shared for each stage, as is the case in typical commercial lock-in amplifiers. Then, the delay time $T_{D_{i}}$ and rise time $T_{R_{i}}$ for the i-th stage is equivalent to $\tau_{1}$ and $\tau_{1}\sqrt{2\pi }$, respectively. As shown in Figure 3, the settling time can be defined as the sum of the delay time and half rise time. From Equations (4) and (5), the settling time for the *m*-th order LPF stage is calculated as follows:

(8) $$\begin{array}{cc} T_{S}=T_{D}+\alpha \left(\gamma \right)\frac{T_{R}}{2} & =mT_{D_{1}}+\frac{\alpha \left(\gamma \right)}{2}{\left(\overset{m}{\underset{i=1}{\sum }}T_{R_{i}}^{2}\right)}^{\frac{1}{2}}\\ & =\left(m+\alpha \left(\gamma \right)\sqrt{\frac{m\pi }{2}}\right)\tau_{1}.\\ & =\tau_{1}/\beta \left(m,\;\gamma \right), \end{array}$$

where β(m, γ) is defined by

(9) $$\beta \;\left(m,\;\gamma \right)={\left(m+\alpha \left(\gamma \right)\sqrt{\frac{m\pi }{2}}\right)}^{-1}.$$

For 90% and 95% settling time, α≈1.3 and α≈1.6 are respectively determined from $\alpha \left(\gamma \right)=\sqrt{2}{\mathrm{erf}}^{-1}\left(2\gamma -1\right)$. From Equation (6), $\tau_{1}$ needs to be limited to obtain the signal level at γ% before the tip starts to be off the surface:

(10) $$\tau_{1}\leq \beta \left(m,\;\gamma \right)DT.$$

In addition, $\tau_{1}$ should satisfy the next condition equivalent to Equation (7), because the signal also needs to increase up to the required level before the tip moves to the location corresponding to the next pixel of the image, as follows:

(11) $$\tau_{1}\leq \beta \left(m,\;\gamma \right)T_{p}.$$

Since we can assume $T\leq T_{p}$ in a normal operation, if $\tau_{1}$ satisfies the first condition, it also satisfies the second one. The second condition is utilized for obtaining the S/N ratio in the conventional PFT-SNDM.

In order to calculate the S/N ratio, we consider the noise level at the output of the lock-in amplifier. Here, we assume that noise density is constant within the measurement bandwidth and does not change regardless of whether the tip is in contact with the surface or not. The −3 dB cut-off frequency or the measurement bandwidth is connected to the rise time by the following relationship:

(12) $$B_{-3\mathrm{dB}}\approx \frac{\zeta \left(\gamma \right)}{T_{R}}.$$

Here, ζ denotes a constant depending on γ. For γ=90% and 95%, ζ≈0.34 and 0.43, respectively. In addition, the equivalent noise bandwidth $B_{N}$ is determined by $B_{-3\mathrm{dB}}$.

(13) $$B_{N}\approx \xi \left(m\right)B_{-3\mathrm{dB}},$$

where ξ is a constant determined by the order of the LPF stage. For the m-th order detector, we obtain more specific representation, as follows:

(14) $$B_{N}\approx \frac{\zeta \left(\gamma \right)}{\tau_{1}\sqrt{2m\pi }}\xi \left(m\right).$$

Then, we give the S/N ratio at the output of the lock-in amplifier by

(15) $$\left(\frac{S}{N}\right)=\frac{\gamma S}{n\sqrt{B_{N}}}\approx \frac{\gamma S}{n}\sqrt{\frac{\sqrt{2m\pi }}{\zeta \left(\gamma \right)\xi \left(m\right)}}\sqrt{\tau_{1}}$$

Here, S denotes the maximum intensity of the d*C*/d*V* signal at a given location or pixel. For the m-th order LPF stage, we obtain the following representation from Equation (10):

(16) $$\left(\frac{S}{N}\right)\leq \frac{\gamma \kappa \left(m,\;\gamma \right)S}{n}\sqrt{DT},\;$$

where κ denotes a constant determined by a given m and γ as follows:

(17) $$\kappa \left(m,\;\gamma \right)=\sqrt{\frac{\sqrt{2m\pi }\beta \left(m,\;\gamma \right)}{\zeta \left(\gamma \right)\xi \left(m\right)}}$$

The right-hand side of Equation (16) is obtained for $\tau_{1}=\beta DT$ and gives the achievable S/N ratio at the output of the lock-in amplifier. In BA-PFT-SNDM, the output of the lock-in amplifier is further averaged over the number of the contact cycles per one pixel to give the d*C*/d*V* signal at the pixel. Since the average number of the samples per one pixel can be given by $T_{p}/T$, the averaging reduces the noise by a factor of $\sqrt{T_{p}/T}$. This implies that the equivalent noise bandwidth for BA-PFT-SNDM is given by $B_{N,\mathrm{BA}}=B_{N}/\left(T_{p}/T\right)$. Then, the S/N ratio of BA-PF-SNDM is described as follows:

(18) $${\left(\frac{S}{N}\right)}_{\mathrm{BA}}\leq \frac{\gamma \kappa \left(m,\;\gamma \right)S}{n}\sqrt{DT_{p}}.$$

For the conventional PFT-SNDM, because of signal reduction by a factor of D, the S/N ratio is given by

(19) $${\left(\frac{S}{N}\right)}_{\mathrm{PFT}}=\frac{\gamma DS}{n\sqrt{B_{N,\mathrm{PFT}}}}.$$

$B_{N,\mathrm{PFT}}$ denotes the equivalent noise bandwidth of PFT-SNDM. For obtaining the S/N ratio improvement rate (SNIR), the filter order is assumed to be the same both in BA-PFT-SNDM and PFT-SNDM. Since $B_{N,\mathrm{PFT}}$ is determined by $\tau_{1}$ satisfying Equation (11) in PFT-SNDM, the S/N ratio is described as follows:

(20) $${\left(\frac{S}{N}\right)}_{\mathrm{PFT}}\leq \frac{\gamma \kappa \left(m,\;\gamma \right)DS}{n}\sqrt{T_{p}}.$$

The achievable S/N ratio is given by the right-hand side in Equation (20) derived from $\tau_{1}=\beta T_{p}.$ It is noted that Equations (19) and (20) are derived under the assumption that $\tau_{1}$ is comparable or higher than T, which implies that the output of the lock-in amplifier to the input pulse train can be well approximated by the step response with the input height equivalent to D (See, Appendix A). This assumption is normally satisfied for PFT-SNDM, because $\tau_{1}$ is set to be higher or equivalent to T. Equations (18) and (20) yield the achievable SNIR as follows:

(21) $${\left(\mathrm{SNIR}\right)}_{\mathrm{BA}/\mathrm{PFT}\;}=\frac{1}{\sqrt{D}}$$

Therefore, BA-PFT-SNDM can improve the S/N ratio by a factor of 1/$\sqrt{D}$, as mentioned in the previous brief report [29]. Equation (20) for D=1 yields the S/N ratio for C-SNDM:

(22) $${\left(\frac{S}{N}\right)}_{C}\leq \frac{\gamma \kappa \left(m,\;\gamma \right)S}{n}\sqrt{T_{p}}$$

By comparing Equation (22) to Equation (16), the S/N ratio in BA-PFT-SNDM is equivalent to that in C-SNDM, staying $DT_{p}$ for one pixel rather than $T_{p}$, which results from the fact that the achievable S/N ratio is essentially determined by the total contact time for a given measurement time under the assumptions here. In conclusion, the achievable S/N ratio is highest in C-SNDM, followed by BA-PFT-SNDM and the conventional PFT-SNDM in order, with a ratio of $1:\sqrt{D}:D$ for a given measurement time.

**Figure 4:**
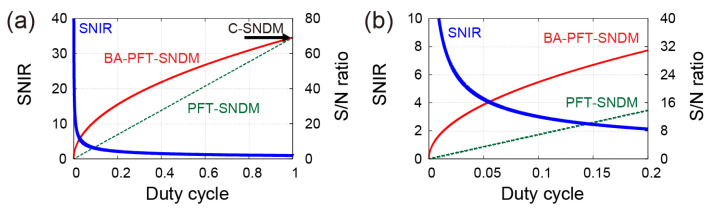
SNIR and comparison of S/N ratios in BA-PFT-SNDM and PFT-SNDM under typical measurement parameters. (**b**) magnifies (**a**) in the range of duty cycle from 0 to 0.2.

**Figure 5:**
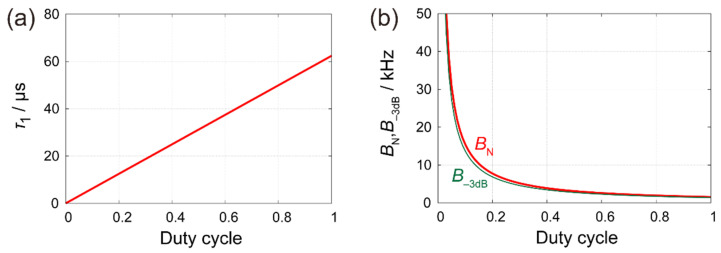
Time constant and bandwidth of the LPF stage at the lock-in amplifier to obtain the highest signal level in BA-PFT-SNDM. (**a**) Time constant of the LPF per one-order (**b**) −3 dB cut-off (green curve) and equivalent noise bandwidth (red curve).

Figure 4 shows SNIR by BA-PFT-SNDM and the comparison of S/N ratios as a function of duty cycle under a typical condition. Here, we assume $T=0.5\;\mathrm{ms},\;\;T_{p}=4.0\;\mathrm{ms},\;S=$ 0.3 aF, and n=0.3 zF/$\sqrt{\mathrm{Hz}}$. In addition, we take the signal at the settling level as γ=95% and m=4, which yields α(95%)≈1.6, ξ(4)≈1.1 and ζ(95%)=0.43. The achievable SNIR is shown by a blue thick solid curve, which is inversely proportional to the square root of the duty cycle. As given by Equation (21), the achievable SNIR is independent of specific parameters except duty cycle and rapidly increases with the decrease of the duty cycle. Figure 5 shows the dependence of $\tau_{1}$, $B_{N}$, and $B_{-3\mathrm{dB}}$ on the duty cycle to obtain the highest signal level at γ=95% settling time. For D=0.1 and m=4, $\tau_{1}$ needs to be less than 6.3 μs, which results in the wider detection bandwidth of $B_{-3\mathrm{dB}}=$14 kHz corresponding to $B_{N}=15$ kHz at the LPF stage of the lock-in amplifier. At the final stage of signal acquisition, we can average eight samples per one pixel of the image, because the average number of contacts for one pixel is obtained from T=0.5 ms and $T_{p}=4.0\;\mathrm{ms}$. Then, the total equivalent noise bandwidth is to be $B_{N,\mathrm{BA}}\approx 1$.9 kHz.

## 4. Experimental Demonstration

**Figure 6:**
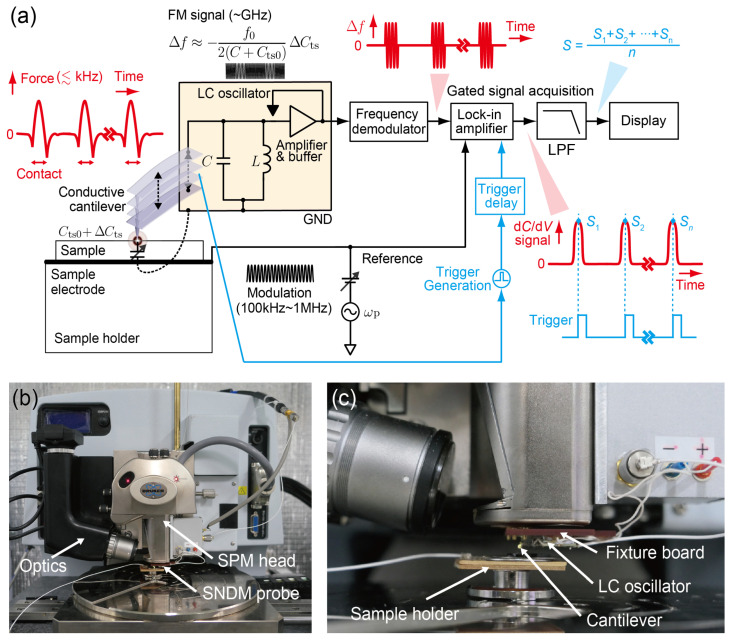
(**a**) Schematic diagram of BA-PFT-SNDM. Gated signal acquisition is realized by triggered signal sampling at the lock-in amplifier. The generated trigger pulse train is synchronized with periodic cantilever motion and appropriately delayed to obtain the highest signal intensity. The signal is further averaged by offline software averaging. (**b**) Overview photograph of the SPM system (Bruker Dimension Icon) with a SNDM probe. (**c**) Close-up picture of the SNDM probe attached to the SPM head.

We experimentally demonstrate that BA-PFT-SNDM achieves a higher S/N ratio than PFT-SNDM. Figure 6a shows a schematic diagram of BA-PFT-SNDM. We realized the proposed scheme by combining a commercial scanning probe microscopy system (Bruker Dimension Icon, Billerica, MA, USA) operating in PFT-AFM, a frequency demodulator (Anritsu MS616B, Atsugi, Japan), a lock-in amplifier (Zurich Instruments HF2-LI, Zurich, Switzerland) with a triggered (gated) sampling mode, a function generator (NF Corp. WF1948, Yokohama, Japan) generating the triggers, and a lab-made software offline signal averager. A trigger pulse train was generated by utilizing the function generator synchronized with the cantilever excitation signal available from the SPM system. For signal conditioning, we additionally inserted a band-pass filter (NF Corp. MF3611, Yokohama, Japan) between the function generator and the SPM system. The pulse train was then appropriately delayed for triggering the data sampling by the lock-in amplifier at the timing of the highest signal from the frequency demodulator. The data were stored in a personal computer and processed by the lab-made software created by Python language.

For demonstration, we measured a test Si sample, which had patterned p- and n-, n^+^ doped areas [38]. We mounted a micro-cantilever coated with Pt-Ir (Nanosensors PPP-EFM, Neuchâtel, Switzerland) with a nominal force constant of 2.8 N/m and resonance frequency of 75 kHz on a SNDM probe oscillating at about 1 GHz. The SNDM probe was specially designed in our laboratory and can be attached to the scanner head of the Icon SPM system, as shown in Figure 6b,c. The SNDM probe is made of a custom-made LC oscillator working as a capacitance sensor mounted on a hand-made fixture board. The cantilever was glued on a particular electrode of the LC oscillator with a conductive paste. The cantilever on the SNDM probe can be located at a position compatible with the laser and optical microscopy in the head. The measurement was done in air at room temperature. For comparison, we acquired images with three different modes: C-SNDM, PFT-SNDM, and BA-PFT-SNDM. The PFT- and BA-PFT-SNDM images were obtained simultaneously. Then, a C-SNDM image was taken separately under the same experimental conditions, except the imaging mode. In PFT- and BA-PFT-SNDM, the contact frequency was set at 2 kHz, which is typical for PFT-AFM. We applied a sinusoidal voltage of 0.2 V_pk_ at a frequency of 200 kHz to the test sample. All images were processed by Gwyddion software [39], because the software can handle BA-PFT-SNDM image files saved in a file format different from the Bruker’s standard SPM format. The raw data from BA-PFT-SNDM included time sequences of peak d*C*/d*V* values and corresponding lateral tip positions, or X and Y scan voltages. The peak d*C*/d*V* values were sampled by the HF2LI lock-in amplifier in the triggered sampling mode. Simultaneously, the X and Y monitor voltages were acquired using two auxiliary analog inputs of the lock-in amplifier, which were wired to a signal access module of the Nanoscope V controller. By using the lab-made Python software, the raw-data were converted to the image data in a so-called XYZ text format (‘Z’ is assigned to d*C*/d*V*). Gwyddion can read both XYZ and SPM files and apply a large number of processing functions to the image data in a unified manner.

**Figure 7:**
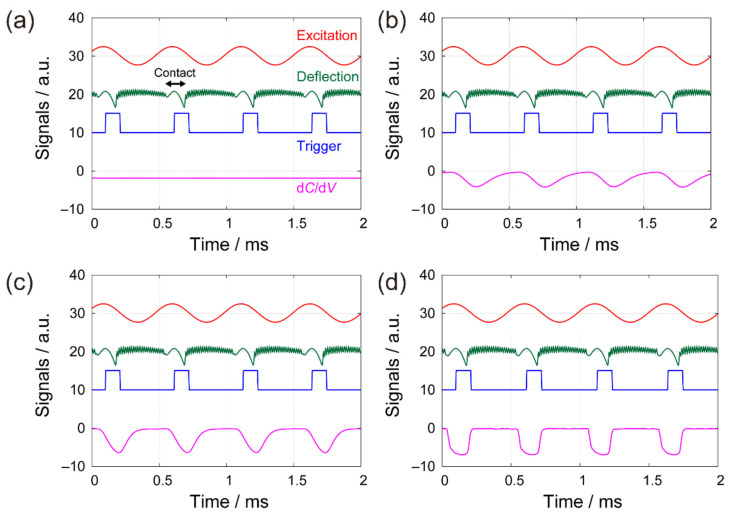
Time series data of the cantilever excitation (solid red curve), cantilever deflection (green), trigger (blue), and d*C*/d*V* signal (pink) for different time constants of the lock-in amplifier (**a**) $\tau_{1}=435\;\mu s$ (**b**) $\tau_{1}=43.5\;\mu s$ (**c**) $\tau_{1}=21.7\;\mu s$ (**d**) $\tau_{1}=4.35\;\mu s$.

Prior to the imaging, we observed the time-series of the signals in PFT-SNDM, keeping the tip lateral position fixed on a specific point of the sample surface to adjust the delay of the trigger and the time constant of the lock-in amplifier. Figure 7 shows the typical time-series data of the cantilever excitation (denoted by a red solid curve), cantilever deflection (green), trigger (blue), and d*C*/d*V* signal (pink) observed for different time constants of the lock-in amplifier by an oscilloscope. As the tip vertically approached and retracted in a periodic manner, it was in intermittent contact with the surface. The cantilever deflection peaked during each contact time and subsequently showed a free oscillation at the natural frequency away from the surface until the contact started again. The trigger signal was synchronized with 2 kHz excitation and deflection signals. The d*C*/d*V* signal showed a negative peak during the contact, which indicates that the tip was located on an n-doping area of the sample. For the time constant much longer than the contact time, the d*C*/d*V* signal had a low constant level because of the averaging effect, as shown in Figure 7a for $\tau_{1}=435\;\mu s$. With lower time constants, the d*C*/d*V* signal exhibited more pronounced negative peaks during the contact, as indicated by Figure 7b for $\tau_{1}=43.5\;\mu s$, Figure 7c for $\tau_{1}=21.7\;\mu s$, and Figure 7d for $\tau_{1}=4.35\;\mu s$. Based on the observation, we could adjust the filter setting of the lock-in amplifier. In addition, we estimated a duty cycle and determined the trigger delay. It is noted that the actual d*C*/d*V* signal had much noise rejected here by an averaging acquisition mode of the oscilloscope to make the preliminary adjustment easier.

**Figure 8:**
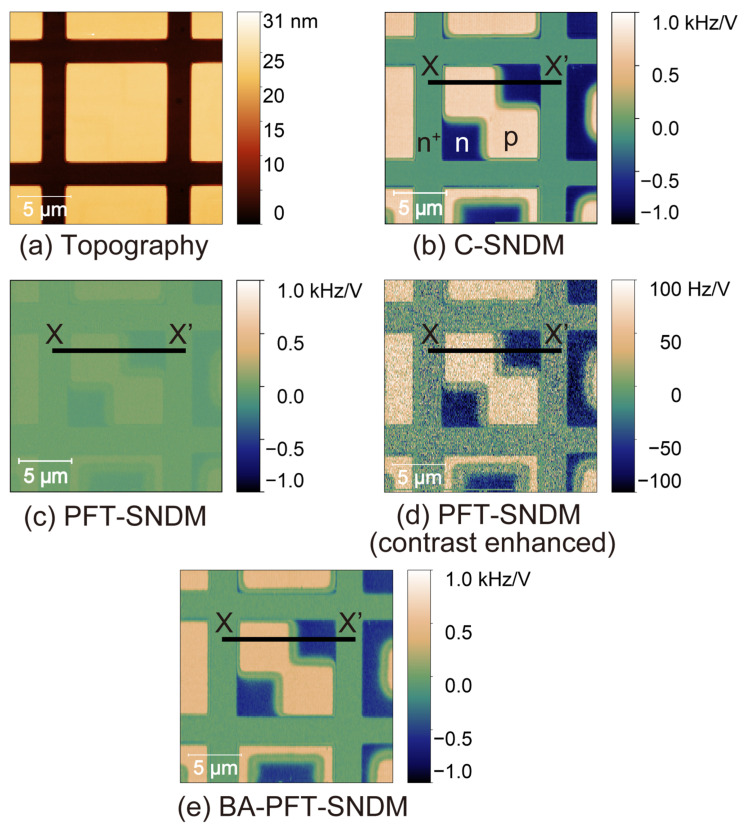
Topographic (**a**), C-SNDM (**b**), PFT-SNDM (**c**,**d**), and BA-PFT-SNDM (**e**) images for the Si test sample with p-, n-, and n^+^ doped areas. (**d**) is a contrast-enhanced image of (**c**). Profiles across the lines from X to X’ in the images are shown in Figure 9.

Figure 8 shows a topographic image (Figure 8a) and d*C*/d*V* images of the Si test sample by C-SNDM (Figure 8b), PFT-SNDM (Figure 8c,d), and BA-PFT-SNDM (Figure 8e). All images were taken with a resolution of 512 × 128 pixels, a scan rate of 0.238 Hz, and measurement time of about 9 min. In these d*C*/d*V* images, n- and p-type areas had negative and positive signals, respectively. Figure 8d is obtained from the same data as Figure 8c but has an enhanced contrast by changing the full range of the color scale. For PFT-SNDM and BA-PFT-SNDM, we determined the contact time and duty ratio to be $T_{c}=62\;\mu s$ and D= 0.12, respectively, based on the preliminary observation of time-series data similar to those in Figure 7. We used the fourth order LPF built in the lock-in amplifier for all imaging modes. The time constant per one order, denoted by $\tau_{1}$ in the previous section, was adjusted to $\tau_{1}=435\;\mu s$ ($B_{N}=180\;\mathrm{Hz}$) for C-SNDM and PFT-SNDM and $\tau_{1}=6.5\;\mu s$ ($B_{N}=12\;\mathrm{kHz}$) for BA-PFT-SNDM. $\tau_{1}$ and $B_{N}$ for BA-PFT-SNDM determined experimentally is in good agreement with the calculated values of $\tau_{1}$ = 7.7 μs and $B_{N}$ = 13 kHz for D=0.12, γ=95%, m=4, and T=512 μs, according to the theory in Section 3. For C-SNDM and PFT-SNDM, the output of the LPF at the lock-in amplifier was input to an external signal acquisition of the SPM controller. As for BA-PFT-SNDM, every peak value of the d*C*/d*V* signal was sampled using the lock-in amplifier under a gated triggered mode and stored as digital data in the personal computer. The stored data of the peak values were further treated by the lab-made software. We obtained an averaged d*C*/d*V* image with 512 × 128 pixels from eight different images, each of which was reconstructed from every eight peak values. This is because the number of peak values per one pixel was eight for the scan rate of 0.238 Hz and the resolution of 512 × 128 pixels. This reduces the bandwidth for BA-PFT-SNDM from 12 to 1.5 kHz, which results in the reduction of noise by a factor of $2\sqrt{2}$. As a result, the predicted increase of the S/N ratio was by a factor of about 2.9. The difference from PFT-SNDM [Figure 8c] shows a much lower signal intensity than C-SNDM (Figure 8b). One can see that the noise is much pronounced when the contrast is enhanced, as shown in Figure 8d. In stark contrast, we found that BA-PFT-SNDM (Figure 8e) exhibits higher signal intensity and improved image quality, as expected.

**Figure 9:**
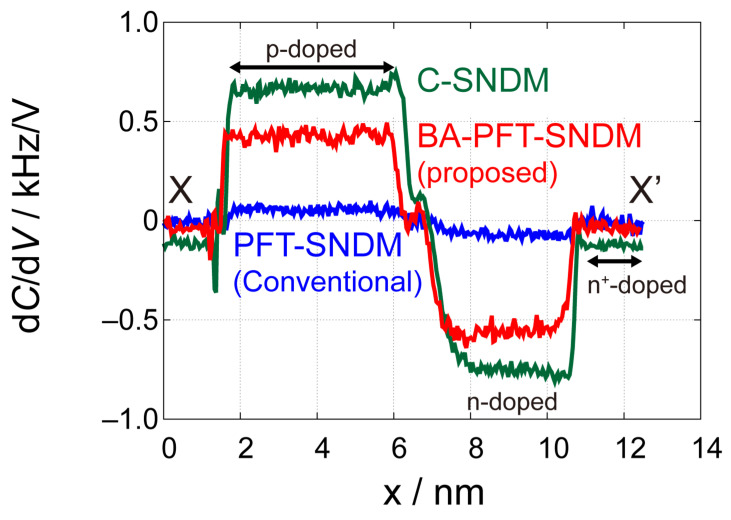
Comparison of the line profiles extracted from the C-SNDM [Figure 8b], PFT-SNDM [Figure 8c], and BA-PFT-SNDM (Figure 8e) images. The profiles are taken along the line from X to X’ in Figure 8.

To discuss the effect of the proposed method quantitatively, we extracted profiles of these d*C*/d*V* images along the lines from X to X’ in Figure 8b–e, as shown in Figure 9. In addition, we compared the signal intensity, noise levels, and S/N ratios among different imaging modes in Table 1 with SNIR. Signal and noise in Table 1 indicate the average of signal intensity and root-mean-squares of signal variations in different areas under the assumption that the fluctuations of dopant concentrations give negligible contribution to the variations of the signal in each area. In comparison to PFT-SNDM, BA-PFT-SNDM showed significantly higher signal intensity on n- and p-type areas, by a factor of 8.3 and 7.9, respectively. These improvement rates of the signal intensity on the n- and p-type areas were in good agreement with the predicted rate of 1/D≈8.3. In addition, we achieved the experimental SNIRs of 4.5 and 4.0 for n and p-type areas, respectively, which are also comparable with the predicted SNIR $1/\sqrt{D}\approx$ 2.9. C-SNDM had the best S/N ratios because of the longest contact time per the given measurement time. SNIRs were 8.3 and 6.5 for p- and n-type areas, respectively, which is also consistent with the predicted value of 1/D=8.3. The SNIRs tended to be smaller than the predicted rates, probably because C-SNDM is more affected by the noise than PFT-SNDM. Although we assumed that the noise level is equivalent regardless of contact, it actually becomes higher when the tip is in contact with the surface. In fact, C-SNDM had higher fluctuation than PFT-SNDM, as observed in Figure 9. As PFT-SNDM had lower contact time, the noise also becomes lower along with the signal intensity. Regarding n^+^-area, there is a discrepancy that cannot be explained by our theory, while BA-PFT-SNDM gives much better results than PFT-SNDM. The signal intensity was improved only by a factor of 5.0, but the noise level was lower than expected. One of the possible reasons is that the d*C*/d*V* signal can include a slight offset on the areas of high dopant concentration, where the signal intensity can become very small, causing errors in the comparison. It is also noted that BA-PFT-SNDM basically had lower signal intensity than C-SNDM, while we assume that BA-PFT-SNDM achieves the same signal intensity as C-SNDM in our theory. We have not identified the cause of the different signal levels, but similar phenomenon is observed in other IC-SNDM using force volume imaging [27]. We speculate that the contact state of the tip on the surface in IC-SNDM is different from that in C-SNDM with much higher lateral forces. Nevertheless, our results demonstrate that BA-PFT-SNDM actually has much higher S/N ratios than the conventional PFT-SNDM in an almost predicted way.

## 5. Application to Imaging Atomically Thin van der Waals Semiconductors

BA-PFT-SNDM is particularly useful for samples prone to the damage. Atomically thin van der Waals materials [40], also called two-dimensional materials, are such materials that can be damaged by C-SNDM [13]. One of the materials is few-layer MoS_2_, which has recently been under intensive research because of the material properties suitable for electronic device applications such as miniaturized transistors [41] and optoelectronics devices [42,43]. However, the electrical characteristics of these ultimately thin materials, consisting almost exclusively of surfaces, are often difficult to control because of the high susceptibility to external influences. One of the important characteristics to be precisely controlled is doping levels on the materials for semiconductor device applications [44,45,46]. However, it is not obvious whether the doping level in an ultra-thin material is the same as that in the bulk counterpart [45].

As already shown by the authors, SNDM is useful for the investigation of the anomalies in the doping levels on atomically thin van der Waals semiconductors [13,14]. Here, we introduce the application of the proposed BA-PFT-SNDM to the measurement of atomically thin natural and Nb-doped MoS_2_. Bulk natural MoS_2_ is typically n-doped. MoS_2_ can also be p-doped by Nb acceptors [47]. We measured ultra-thin natural (SPI Supplies) and Nb-doped MoS_2_ (HQ Graphene) mechanically exfoliated on thermally oxidized Si substrates. The samples were prepared by the so-called Scotch-tape method [48]. We used highly doped Si substrates with a resistivity of 0.001~0.005 Ωcm and a 300 nm-thick thermal oxide layer. We simultaneously obtained PFT-SNDM images as well as BA-PFT-SNDM images. The amplitudes of the applied voltages were 0.5 V_pk_ and 1.0 V_pk_ for natural and Nb-doped MoS_2_, respectively, and the frequency was 200 kHz for both samples.

**Figure 10:**
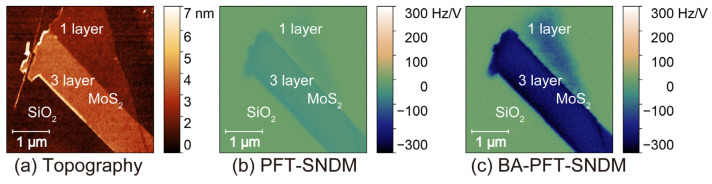
Topographic (**a**), PFT-SNDM (**b**), and BA-PFT-SNDM (**c**) images of few-layer natural MoS_2_ mechanically exfoliated on a thermally oxidized Si substrate.

**Figure 11:**
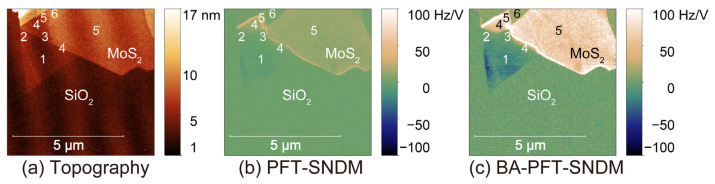
Topographic (**a**), PFT-SNDM (**b**), and BA-PFT-SNDM (**c**) images of atomically thin Nb-doped MoS_2_ mechanically exfoliated on a thermally oxidized Si substrate. Each Arabic number in the figures denotes the number of stacking layers on the corresponding area.

Figure 10 shows topographic (Figure 10a), PFT-SNDM (Figure 10b), and BA-PFT-SNDM images (Figure 10c) for few-layer natural MoS_2_. We determined the number of stacking layers from the topographic heights of the observed MoS_2_ layers. The sample had single- and three-layer MoS_2_ with negative d*C*/d*V* signals, which indicates that these layers were n-doped. In comparison to the PFT-SNDM image (Figure 10b), the BA-PFT-SNDM image (Figure 10c) clearly resolved n-doping on the single-layer area. The results here showed that natural MoS_2_ layers remained n-type semiconductors even in single-layer structures. On the other hand, Nb-doped MoS_2_ showed different characteristics when the number of layers decreased. Figure 11 shows topographic (Figure 11a), PFT-SNDM (Figure 11b), and BA-PFT-SNDM (Figure 11c) images for atomically thin Nb-doped MoS_2_. The Arabic numbers on the images denote the number of stacking layers on the indicated area. One can see that the sample included 1- to 6-layer MoS_2_ layers in the field of view. We could confirm that the 4- to 6-layer MoS_2_ showed positive d*C*/d*V* signals, indicating p-doping on these layers, as expected from Nb-doping. However, as the layer number decreased from 4 to 1, the signal intensity decreased and the polarity changed from positive to negative. We find that this unexpected p- to n-type transition became clearly visible by BA-PFT-SNDM rather than PFT-SNDM, as shown in Figure 11b,c.

It is noted that the unintentional n-doping on MoS_2_ layers has also been reported by different groups recently [45,46]. Siao et al. suggested that sulfur vacancies on the surface layer of MoS_2_ causes n-doping, which is high enough for overcompensating the artificial p-doping on few-layer MoS_2_ [45]. In addition, Fang et al. have reported a p- to n-type transition in few-layer Nb-doped MoS_2_ field effect transistors, with reference to the work by Siao et al. [46]. We think that the enhanced sensitivity of BA-PFT-SNDM established in this paper permitted real-space and nanoscale imaging of the anomalous doping effect on the atomically thin van der Waals semiconductor. The experimental results presented here demonstrate that BA-PFT-SNDM has superior imaging performance to PFT-SNDM. The detailed analysis and discussion on the measurement results are shown in greater detail in Ref. [14]. It is noted that PFT-AFM combined with SNDM has been known for its capability of quantitative nanomechanical mapping such as adhesion, modulus, and dissipation as well as topographic imaging [25]. Our results indicate that BA-PFT-SNDM allows for the possibility of simultaneous nanoelectrical and nanomechanical mapping along with topographic imaging, which will give a clue as to the comprehensive understanding of the material properties.

## 6. Conclusions

In this paper, we proposed BA-PFT-SNDM based on the theory extended here for giving quantitative insights into the S/N ratio of SNDM. In comparison to PFT-SNDM, BA-PFT-SNDM, which is based on gated signal acquisition and averaging, can increase the S/N ratio by a factor of the inverse square root of the duty cycle, or the rate of the contact time to the contact period in PFT-SNDM. Our theory gives the time constant to be chosen for the gated signal acquisition and allows us to predict SNIR in actual BA-PFT-SNDM measurement in a quantitative way. In addition, we experimentally showed that BA-PFT-SNDM improves the S/N ratios at the rate consistent with the predicted SNIR. The SNIR achievable by BA-PFT-SNDM is two to several times in a typical measurement condition. Furthermore, as an application of BA-PFT-SNDM, we presented the imaging of dominant carrier concentration distribution on atomically thin van der Waals semiconductors. We found that, in comparison to PFT-SNDM, BF-PFT-SNDM has significantly higher imaging capability, enabling clear visualization of the p- to n-type transition on few-layer Nb-doped MoS_2_. We believe that the field of SNDM will further extend by the emergence of BA-PFT-SNDM, because it enables damage-less, higher sensitivity, and simultaneous nanoelectrical and nanomechanical imaging even on soft and fragile samples. The idea here can also be applied to the optimization of the S/N ratios in other scanning near-field microwave microscopy such as SMIM [30] operating in peak-force tapping mode [25].

## Figures and Tables

**Table 1:** Signal intensity in Hz_pk_/V_pk_, noise levels in Hz_rms_/V_pk_, and S/N ratios in different imaging modes. SNIRs indicates S/N ratio normalized by those in PFT-SNDM.

	PFT-SNDM			BA-PFT-SNDM				C-SNDM			
Region	Signal	Noise	S/N Ratio	Signal	Noise	S/N Ratio	SNIR	Signal	Noise	S/N Ratio	SNIR
p	52	17	3.1	431	31	14	4.5	662	26	25	8.3
n	−70	17	4.1	−554	34	16	4.0	−748	28	27	6.5
n^+^	−7.4	23	0.32	−37	24	1.5	4.8	−112	14	8.0	25

## Data Availability

The data that support the findings of this study are available from the corresponding author upon reasonable request.

## Appendix A

In BA-PFT-SNDM, where the individual peaks of the d*C*/d*V* signal are acquired with a lower time constant of the LPF stage, only the response to the step input needs to be considered to obtain the S/N ratio given to one pixel of the d*C*/d*V* image. On the other hand, in PFT-SNDM, strictly speaking, we need to consider the response to an input pulse train rather than the single step input. This is because the time constant of the LPF in the lock-in amplifier, denoted by $\tau_{1}$ in Section 3, is normally set to be comparable with or higher than the contact time. As a result, the response of the LPF becomes much slower than the contact time (even if $\tau_{1}∼T$, due to the high order of the LPF in typical conditions). However, the response to the pulse train can still be approximated by a step response with a height of D as follows.

In general, the output Y(s) to the input U(s) is connected by the transfer function G(s) of the LPF stage:

(A1) Y(s)=G(s)U(s).

For the pulse train of the d*C*/d*V* signal starting at t=0, U(s) is described by

(A2) $$U\left(s\right)=\frac{1-e^{-DTs}}{1-e^{-Ts}}\cdot \frac{1}{s}.$$

Thus, from Equations (A1) and (A2), the output can be given by

(A3) $$Y\left(s\right)=G\left(s\right)\;\frac{1-e^{-DTs}}{1-e^{-Ts}}\cdot \frac{1}{s}.$$

Here, let us assume $\tau_{1}∼T$ or $\tau_{1}>T$. This implies that |G(s)|≈1 for |s|≪1/T and |G(s)|≪1 for |s|∼1/T or |s|>1/T is especially well satisfied in the high order LPF. In other words, the LPF passes only s satisfying |s|≪1/T to the output, which allows for the following approximation of U(s) within |s|≪1/T:

(A4) $$Y\left(s\right)=G\left(s\right)\frac{-DTs\left\{1-\frac{DTs}{2!}+\frac{{\left(DTs\right)}^{2}}{3!}-\cdots \right\}}{-Ts\left\{1-\frac{Ts}{2!}+\frac{{\left(Ts\right)}^{2}}{3!}-\cdots \right\}}\cdot \frac{1}{s}\approx G\left(s\right)\frac{D}{s}$$

Equation (A4) implies that the response to the pulse train is approximated by the response to the step input starting at t=0 in a typical measurement setup of PFT-SNDM.
